# Aging RNA granule dynamics in neurodegeneration

**DOI:** 10.3389/fmolb.2022.991641

**Published:** 2022-09-16

**Authors:** Kevin Rhine, Norah Al-Azzam, Tao Yu, Gene W. Yeo

**Affiliations:** ^1^ Department of Cellular and Molecular Medicine, University of California, San Diego, San Diego, CA, United States; ^2^ Stem Cell Program, University of California, San Diego, San Diego, CA, United States; ^3^ Institute for Genomic Medicine, University of California, San Diego, San Diego, CA, United States; ^4^ Neurosciences Graduate Program, University of California, San Diego, San Diego, CA, United States

**Keywords:** neurodegeneration, RNA granules, stress granules, RNA, liquid-liquid phase separation

## Abstract

Disordered RNA-binding proteins and repetitive RNA sequences are the main genetic causes of several neurodegenerative diseases, including amyotrophic lateral sclerosis and Huntington’s disease. Importantly, these components also seed the formation of cytoplasmic liquid-like granules, like stress granules and P bodies. Emerging evidence demonstrates that healthy granules formed via liquid-liquid phase separation can mature into solid- or gel-like inclusions that persist within the cell. These solidified inclusions are a precursor to the aggregates identified in patients, demonstrating that dysregulation of RNA granule biology is an important component of neurodegeneration. Here, we review recent literature highlighting how RNA molecules seed proteinaceous granules, the mechanisms of healthy turnover of RNA granules in cells, which biophysical properties underly a transition to solid- or gel-like material states, and why persistent granules disrupt the cellular homeostasis of neurons. We also identify various methods that will illuminate the contributions of disordered proteins and RNAs to neurodegeneration in ongoing research efforts.

## Introduction

The compartmentalization of cellular contents into membrane-bound organelles is a long-established paradigm in biology. However, recent evidence demonstrates that lipid membranes are not strictly required to segregate macromolecules into distinct structures. Instead, discrete liquid phases—like oil in water—may form when proteins containing intrinsically disordered regions (IDRs) bind to RNA, forming multimeric complexes. These multimeric complexes eventually support a phase transition, in which a dense proteinaceous granule forms by liquid-liquid phase separation (LLPS). A variety of cytoplasmic liquid-like granules form via LLPS, including stress granules (SGs), P bodies, G bodies, and others (Banani et al., 2017). Importantly, many of these granules are seeded in response to stress, and they are disassembled once the stress event ends (Buchan et al., 2008; Wheeler et al., 2016; Jin et al., 2017). However, a granule may persist beyond the stress event if it undergoes a material transition into a solid- or gel-like status (Zhang et al., 2019; Lu et al., 2021). When granules mature into gels, they have reduced interactions with the surrounding cellular milieu and can further transition into fibrils or other aggregated structures (Patel et al., 2015). Solid- or gel-like granules are disastrous for neurons, most of which are not replenished throughout an organism’s lifetime (Lim and Yue, 2015). Dysregulation of the material state of cellular granules is linked with a variety of neurodegenerative diseases, including amyotrophic lateral sclerosis (ALS), Huntington’s, Alzheimer’s, and others (Patel et al., 2015; Li et al., 2016; Wegmann et al., 2018).

RNA is a critical component of phase-separated granules. Many proteins that are enriched within granules contain not only IDRs but also canonical RNA-binding domains (Markmiller et al., 2018). These RNA-binding proteins (RBPs) can therefore multimerize with themselves via IDRs and RNA via their RNA-binding domains (Rhine et al., 2020b). Biochemical studies demonstrate that RNA can reduce the amount of protein needed to form proteinaceous droplets *in vitro* (Kato et al., 2012; Molliex et al., 2015; Niaki et al., 2020; Yang et al., 2020), and elegant cellular experiments are consistent with the idea that cytoplasmic RNA promotes LLPS (Fuller et al., 2020; Bauer et al., 2022). Conversely, an overabundance of RNA may buffer phase separation by diluting multimeric interactions among many RNA and RBP molecules, though this phenomenon is more pronounced in the nucleus (Maharana et al., 2018). The cytoplasmic concentration of RNA can be tuned by stress events, especially translational arrest at polysomes that cause an acute increase in available RNA for RBP binding (Bounedjah et al., 2014; Iserman et al., 2020). In general, longer RNAs are more effectively recruited into granules, but biases toward certain sequence or structure motifs heavily depend on the RBP recognizing the RNA molecule and other polymers in the cell (Khong et al., 2017; Hallegger et al., 2021; Rhine et al., 2022a). Repetitive RNAs may also contribute to granule formation independently of proteins by undergoing self-associations like those found in G-quadruplexes (Boeynaems et al., 2019).

Aside from promoting granule formation, RNA also alters the viscoelastic properties of granules (Roden and Gladfelter, 2021; Laghmach et al., 2022). As mentioned above, RNA promotes RBP multimerization by acting as a scaffold to which proteins may bind (Schwartz et al., 2013; Rhine et al., 2020a). Scaffolds naturally stabilize the resulting condensate (Decker et al., 2022; Sanchez-Burgos et al., 2022), which can accelerate coarsening into solid- or gel-like material states (Bose et al., 2022). In addition, the physical interaction between RNA and its cognate proteins may lead to conformational rearrangements in the protein that shield certain domains, inhibiting efficient dissolution by chaperones that resolve granules (Yoshizawa et al., 2018). Finally, mutations in RBPs can further promote solid-like transitions (Zhu et al., 2014; Niaki et al., 2020). Indeed, many mutations identified in ALS affect RBPs containing IDRs, including FUS, TDP-43, hnRNPA1, and others (Sreedharan et al., 2008; Vance et al., 2009; Kim et al., 2013). RNA promotes condensation of all these proteins, indirectly supporting an aberrant material state transition consistent with inclusions found in neurodegeneration patients.

Therefore, RNA has a critical role in establishing the biophysical properties of condensates. Given the vast number of biological processes that require RNA, sequestration of RNA into disease-associated granules is an inherently perturbative outcome that destabilizes cellular homeostasis and eventually promotes cell death (Pushpalatha et al., 2022). In the following sections, we review the various regulatory and biophysical processes that contribute to RNA granule aging, especially in the context of neurodegeneration as persistent RNA granules disrupt cellular homeostasis. We also present various techniques that may be used to further our understanding of how RNA impacts granule maturation.

### Turnover of stress granules in health and disease

**FIGURE 1:**
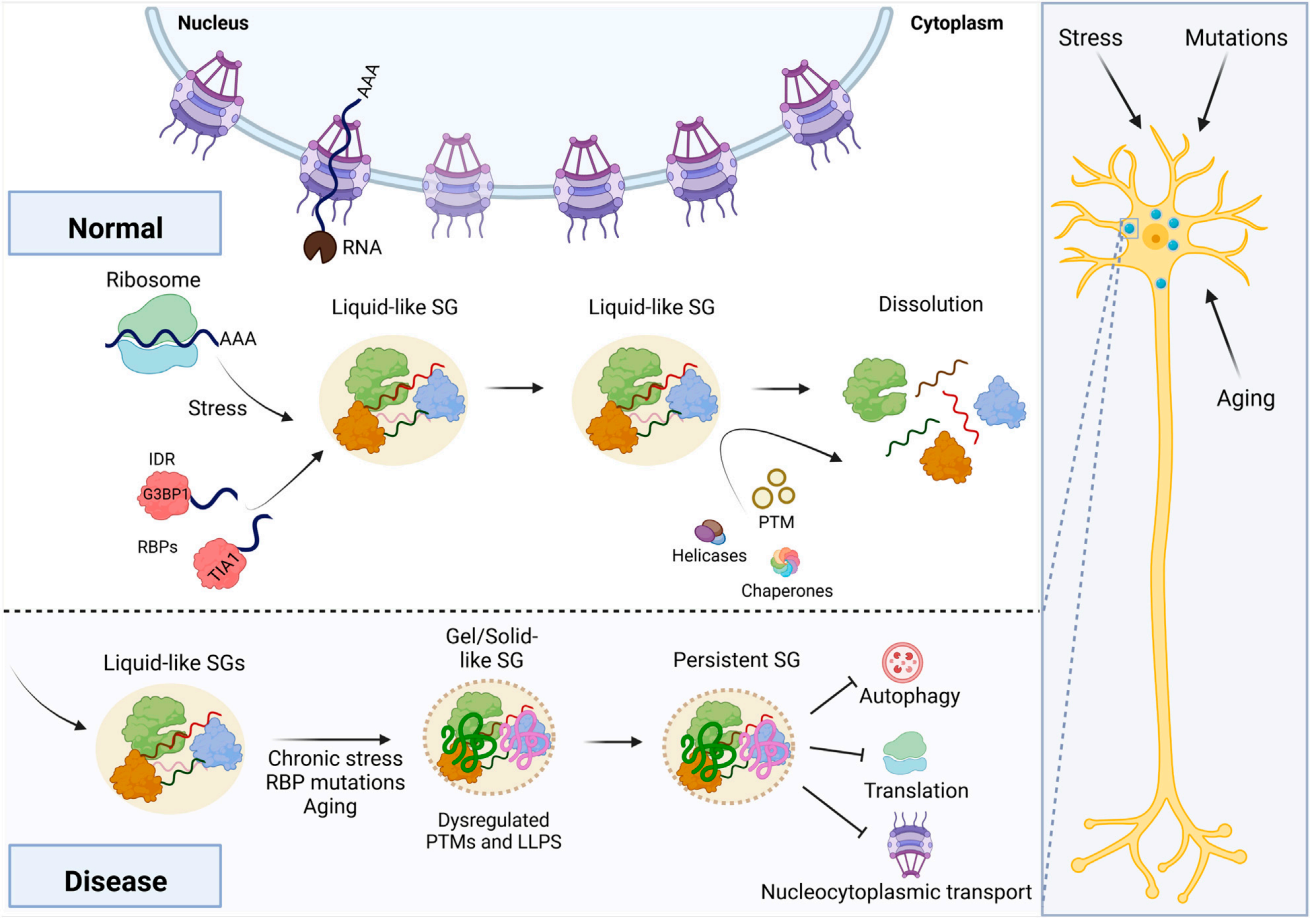
Maturation of RNA granules into disease-associated states. Stress, aging, and mutations can all stall neuronal translation, initiating stress granule assembly via G3BP1. Chronic stress exposure or other mechanisms may lead to a disease-associated outcome, in which the granule transitions to a gel- or solid-like state. In turn, this persistent granule disrupts cellular homeostasis, including autophagy, translation, and nucleocytoplasmic transport. This figure was created using BioRender.

The normal life cycle of a stress granule involves rapid formation and quick dissolution regulated by several mechanisms. Upon different stress conditions, multiple pathways including inhibition of mTOR, phosphorylation of eIF2a and disruption of eIF4F complex induce SG formation (Harding et al., 2000; Gilks et al., 2004; Thedieck et al., 2013). All three pathways converge to stall and disassemble polysomes, which is arguably the common trigger of SG formation (Buchan and Parker, 2009). When SGs form, RBPs and mRNA molecules play a major role. Two RBPs, in particular, have been thoroughly studied for the assembly of cytoplasmic SG: T-cell intracellular antigen 1 (TIA-1) and Ras-GTPase-activating protein SH3-domain-binding protein 1 (G3BP1) (Tourriere et al., 2003; Gilks et al., 2004). G3BP1 and its homolog G3BP2 are the core components of stress granules (Figure 1), which undergo RNA-dependent LLPS by sequestering free RNA to generate protein-RNA condensates (Guillen-Boixet et al., 2020; Yang et al., 2020). G3BP1 can also interact with many IDR-containing RBPs, including Caprin-1 and TIA-1, and both promote G3BP1/2-mediated LLPS (Guillen-Boixet et al., 2020; Yang et al., 2020). The size and liquid properties of RNA-protein condensates in cells are affected by RNA recruitment (Garcia-Jove Navarro et al., 2019; Roden and Gladfelter, 2021). Furthermore, the availability of RNA–RNA assemblies is important for granule assembly *in vitro* (Van Treeck et al., 2018). Trcek *et al.* demonstrated in *Drosophila* germ cells that different mRNA features govern mRNA localization to granules and self-assembly within granules; they noted that localization is encoded by specific RNA regions, whereas self-assembly is RNA sequence-independent (Trcek et al., 2020).

Canonical RNA turnover in SGs occurs in a stepwise manner (Wheeler et al., 2016). First, translation is slowed during stress, and polysomes release their respective messenger ribonucleoproteins (mRNPs). The free mRNP particles oligomerize via protein-protein, protein–RNA, or RNA–RNA interactions. When more RNAs enter the non-translating pool, oligomers form stable core assemblies (Decker et al., 2022). As a result of fusion and mRNP recruitment, a mature SG with a distinct core-shell substructure is generated. Even mature SGs (especially the “shell”) are still in dynamic equilibrium, exchanging materials with polysomes and P bodies (Wheeler et al., 2016). It is worth noting that a recent super-resolution imaging study showcased comparable translation efficiency of their reporter in SGs over outside of SGs, suggesting that the SG environment is not inhibitory to translation and that global translation inhibition in response to stress is more likely to be upstream of rather than the consequence of SG formation (Mateju et al., 2020). SGs begin to break down via shell loss followed by core dispersal, and mRNPs re-enter translation. Translationally-stalled mRNAs are titrated out of SGs, producing structural instability in the protein complexes and eventually gradual deconstruction of the visible SGs (Wheeler et al., 2016). Several lines of evidence have shown that RNA can mitigate excessive protein-protein interactions, which lead to pathological aggregates seen in a variety of neurodegenerative disorders (Maharana et al., 2018; Mann et al., 2019; Zacco et al., 2019), emphasizing the critical balance between protein-protein, RNA–protein, and RNA–RNA interactions required for proper SG assembly and disassembly. Post-translational modifications also play a critical role in SG disassembly, Maxwell *et al.* revealed an important function of heat-induced polyubiquitylation, which is instrumental in preparing cells for the restart of cellular activities upon stress release (Maxwell et al., 2021). Furthermore, the SG scaffold protein G3BP1 is a key substrate for polyubiquitin-dependent disassembly of heat-induced SGs (Gwon et al., 2021). One crucial mechanism in this recovery phase is the restart of translation, which is accompanied by the ubiquitin-dependent disassembly of SGs (Maxwell et al., 2021). Lastly, increased recruitment of small ubiquitin-like modifier ligases into the SGs is observed upon stress exposure leading to SUMOylation of proteins necessary for SG disassembly (Marmor-Kollet et al., 2020).

RNA helicases also have a major role in RNA granule dynamics and could contribute to the interconnection between mRNA storage, translation, and decay (Hondele et al., 2019; Weis and Hondele, 2022). Recent work shows that assembly and disassembly of RNA granules are monitored by DEAD-Box helicase 6 (DDX6), an essential P body component, in neuronal maturation both *in vitro* and *in vivo* (Bauer et al., 2022). ATP, which is consumed by RNA helicases, is required for the fast construction, remodeling, and disassembly of stress granule components (Wolozin and Ivanov, 2019). Furthermore, DDX6 granule condensation requires Staufen-2-dependent RNA interaction during synaptic inhibition. This is most likely owing to Stau2’s participation in mRNA transport and redistribution (Bauer et al., 2022). In addition, recent work has shown that sex chromosome-encoded RNA helicases such as DDX3X and DDX3Y influence the formation of RNA granules in an ATP-independent manner but have different LLPS propensities (Shen H. et al., 2022).

Nuclear import of granule-associated proteins is another important layer of SG regulation (Guo et al., 2018), and defects in nucleocytoplasmic transport are a significant pathogenic factor in ALS (Zhang et al., 2015; Gleixner et al., 2022). Cytoplasmic protein clumps, which are frequently observed in many neurodegenerative illnesses, impair nucleocytoplasmic transport, implying that abnormalities in nucleocytoplasmic transport might constitute a general cause of neurodegeneration (Hutten and Dormann, 2020; Gonzalez et al., 2021; Odeh et al., 2022). Critical nucleocytoplasmic transport components such as karyopherins (importins and exportins), Ran GTPase, and nucleoporins translocate to stress granules in response to cellular stress, resulting in inefficient nucleocytoplasmic transport (Zhang et al., 2018). Importantly, in C9ORF72 ALS models, blocking stress granule construction decreases these abnormalities as well as neurodegeneration (Zhang et al., 2018). These discoveries linked two pathophysiological processes, stress granule construction and nucleocytoplasmic transport disruption, into a single pathway that leads to pathogenesis. Thus, an intriguing issue of the present work is analyzing how changes to essential stress granule components or remodeling machinery affect the various phases of this assembly and disassembly process.

### RNA promotes the maturation of granules to solid-like material states

Following granule formation, the cell has a variety of mechanisms to promote dissolution of granules, including posttranslational modifications that signal for turnover of critical RBPs, expression of chaperones that recognize granule proteins, and helicase activity that ejects RBPs from RNA (Guo et al., 2018; Hondele et al., 2019; Maxwell et al., 2021). However, complete resolution of RNA granules may not be possible if the granule has coarsened into a solid- or gel-like state (Figure 1). Many *in vitro* studies demonstrate the effect of aging, but there are two separate biophysical processes through which a granule may achieve a solid- or gel-like state. A recent study by Jawerth *et al.* proposed that condensates do not become gels *per se* and instead behave as a Maxwell fluid with strongly increasing viscosity as a function of condensate age (Jawerth et al., 2020). Other studies indicate that RBPs bound to RNA may undergo dynamical arrest via percolation, halting exchange with the surrounding dilute phase (Harmon et al., 2017; Rhine et al., 2020a; Choi et al., 2020; Bose et al., 2022; Linsenmeier et al., 2022; Mittag and Pappu, 2022). Granules may also partially transition to gels, as was observed with the RBP FUS (Shen Y. et al., 2022). In principle, these mechanisms likely arrive at the same outcome: an RNA granule that is resistant to dissolution because of a change in its material properties.

Many neurodegenerative diseases are linked to the formation of fibrils or solid-like inclusions (Kim et al., 2013; Nomura et al., 2014; Bowden and Dormann, 2016; French et al., 2019), which have similar material properties to aged RNA-containing SGs (Zhu et al., 2014; Patel et al., 2015; Murray et al., 2017; Fonda et al., 2021). Proteins associated with ALS (FUS, TDP-43, hnRNPA1, etc.), Alzheimer’s (Tau), and Parkinson’s (α-synuclein) undergo both LLPS and aggregation into fibers (Gotz et al., 2001; Roberson et al., 2007; Sreedharan et al., 2008; Vance et al., 2009; Kim et al., 2013; Wegmann et al., 2018; Ray et al., 2020). Although fibers are thought to be the causative agent of disease, certain ALS-associated RBPs incorporate into liquid-like stress granules (Bentmann et al., 2012; Markmiller et al., 2018; Reber et al., 2021; An et al., 2022). ALS-linked mutations in these RBPs accelerate the granule aging process or inhibit recognition by protein chaperones (Guo et al., 2018; Hofweber et al., 2018; Niaki et al., 2020). Aged RNA granules can be deleterious on their own by stalling translation via RNA sequestration and by preventing cellular recovery from stress (Reineke and Neilson, 2019; Pushpalatha et al., 2022). Disease-linked mutations cause some proteins like TDP-43 to bypass the liquid phase altogether, forming aggregates or fibrils at physiological concentrations (Patel et al., 2015; Cao et al., 2019; Mathieu et al., 2020). In these cases, LLPS may act as a protective agent to prevent or decelerate deleterious solid- or gel-like transitions.

Recent evidence highlights the role of RNA in promoting the transition of granules to solid-like material states. The most common genetic cause of ALS—a repeat expansion of the C9ORF72 locus—leads to the expression of the highly toxic (GGGGCC)_n_ RNA motif and translation of repetitive dipeptide chains, especially poly-RG and poly-PR (DeJesus-Hernandez et al., 2011). Importantly, this RNA repeat engages in multivalent base pairing, promoting granules that transition into a gel-like state (Jain and Vale, 2017). Other disease-associated RNA repeats also impact granule aging *in vitro* (Ma et al., 2022), and the self-association of G-rich RNA forms solid-like fibers (Boeynaems et al., 2019). Normal mRNA sequences may also contribute to RNA self-assembly or gelation, as has been observed in worms and flies (Lee et al., 2020; Trcek et al., 2020). It is possible that P-body-associated RNA helicases are required for proper resolution of multivalent RNA tangles (Hondele et al., 2019; Majerciak et al., 2021; Linsenmeier et al., 2022), which can age condensates into gels. Together, these studies demonstrate that RNA can promote solid- or gel-like transitions in RNA granules, and sequencing-based technologies like CLIP-seq and others may help identify changes in RNA biology during disease (Van Nostrand et al., 2016; Hallegger et al., 2021; Wollny et al., 2022) (Table 1). Although we do not yet know how exactly aged RNA granules and fibers lead to cell death, the next section will discuss how the persistence of aged granules and fibers may disrupt cellular homeostasis.

**TABLE 1:** Imaging, sequencing, and biochemical methods for studying RNA granules.

	Method	Approach	Advantage	Disadvantage	References
Imaging techniques	Immunofluorescence/tagging with fluorescent protein	Colocalization with granule markers	Technically straightforward; quantitative measurement of partition coefficients	Low throughput, requires prior knowledge of granule proteins, use of fixatives may alter granule properties	Irgen-Gioro et al. (2022)
	Single particle/molecule tracking (SPT/SMT)	Substoichiometric labeling of proteins with photostable fluorophores for single-particle tracking *in vivo*	Compatible with IF/tagging methods above; live imaging under various conditions	Requires super resolution imaging, labeling; may disrupt the localization of the original transcripts or proteins	(Li et al., 2016; Horvathova et al., 2017; Mateju et al., 2020; Moon et al., 2020)
	RNA-fluorescence *in situ* hybridization (FISH)/Single-molecule FISH (smFISH)	Hybridization-based method to label RNAs with fluorescent probes	Compatible with IF, quantitative, feasible to multiplex	Costly, require prior knowledge, use of fixative may alter granule properties	(Ivanov et al., 2011; Zurla et al., 2011; Khong et al., 2017)
Omics techniques	APEX proximity labeling (APEX2)	APEX2 protein fusion labels nearby proteins and RNAs when biotin-phenol and hydrogen peroxide are added	Creates a snapshot of proteins and RNAs in proximity to a protein of interest (suitable for studying dynamics)	Partitioning and diffusion of proteins within stress granules increases noise and background	(Markmiller et al., 2018; Padron et al., 2019; Elmsaouri et al., 2022)
	Bio-ID/TurboID	BirA mutant fused to protein of interest biotinylate the proteins in close proximity in living cells	Accumulated labeling in a period of time (also suitable for transitory interactors)	Partitioning and dynamics of proteins within stress granules increases noise and background, cannot label RNA	(Roux et al., 2012; Kim et al., 2016; Youn et al., 2018)
	CLIP-Seq	Immunoprecipitation of crosslinked RBP-RNA interactions; sequencing of RNA molecules with Illumina methodology or equivalent	Allow base-resolution identification of RBP binding sites on target RNAs, compatible with proximity labeling (Proximity-CLIP)	Requires IP-grade antibodies	(Niranjanakumari et al., 2002; Van Nostrand et al., 2016; Benhalevy et al., 2018)
Biochemical/biophysical techniques	RNP granule purification	Fractionation of RNP granules by ultracentrifugation, followed by immunoprecipitation of granule markers	Compatible with various downstream analyses, including mass spec and RNA-Seq	Loses weakly associated proteins and RNAs in the “shell” of the granule	Matheny et al. (2019)
	Fluorescence recovery after photobleaching (FRAP)	Intense photobleaching of granules; tracking of fluorescence recovery over time	Can help determine viscoelastic properties of granules	Recovery may be due to internal or external diffusion, so the parameters need to be decoupled; bleaching laser apparatus needed	(Ganser and Myong, 2020; Rhine et al., 2022b)
	Microrheology	Beads within granules are used to determine the diffusion within the condensate	Determines the internal diffusion coefficient, which is used to calculate the viscoelastic properties of the granule	Rheology is technically difficult to establish in a cell	Elbaum-Garfinkle et al. (2015)

### Persistent granules disrupt cellular homeostasis and could potentially serve as therapeutic targets

The persistence of RNA granules may disrupt cellular homeostasis through multiple mechanisms, including posing as a cytotoxic stimulus, sequestering proteins important for maintaining cellular homeostasis, and altering SG assembly and disassembly (Figure 1). In Alzheimer’s disease, amyloid-beta aggregates stimulate persistent SGs (Ghosh and Geahlen, 2015), and SG assembly promotes the phosphorylation of tau (Vanderweyde et al., 2012), potentially accelerating the onset of neurodegeneration. In the case of multiple polyglutamine (polyQ) diseases like Huntington’s, the polyQ aggregates could trigger various types of stresses (Labbadia and Morimoto, 2013; Matos et al., 2019), including misfolded protein stress. The polyQ aggregates also sequester SG components (Uchihara et al., 2001; Furukawa et al., 2009; Sleigh et al., 2020), leading to aberrant SG composition. These SGs contain misfolded proteins, further sequestering components of autophagy (Mateju et al., 2017). As one of the SG clearance pathways (Ryu et al., 2014), impaired autophagy could in turn disrupt the disassembly of SGs. In ALS, SGs colocalize with TDP-43 and poly-GR aggregates (Liu-Yesucevitz et al., 2010; Chew et al., 2019), and chronic SGs could directly contribute to ALS onset (Daigle et al., 2016; Zhang et al., 2019). The poly-PR aggregates could directly interact with ribosomes and impair protein translation (Zhang et al., 2018). Global splicing and RNA localization may also be altered due to the sequestration of important regulators in these cellular processes (Charizanis et al., 2012; Markmiller et al., 2021).

The unique formation of persistent granules in these neurodegenerative diseases enables the development of new strategies for therapeutic treatment. antisense oligonucleotides and RNA-targeting CRISPR have been demonstrated to eliminate toxic RNA granules (Lee et al., 2012; Batra et al., 2017) when particular RNAs are known to form these granules. Small molecule drugs could potentially be developed to target specific proteins responsible for the assembly or preventing disassembly of persistent granules. More efforts are needed to better depict the components of these granules under different disease conditions (Table 1). Meanwhile, explorative drug screens could be performed to repurpose existing drugs for resolving persistent granules in different disease contexts (Fang et al., 2019).

### Technologies for investigating RNA granules

Pioneering research has laid the groundwork for our present understanding of RNA granules in neurodegenerative disease, expanding our understanding of the fundamental mechanisms of granule assembly and disassembly, as well as their composition and structural organization. However, there remains many gaps in the field. To conclude, we review new methods to aid our understanding of RNA granules (Table 1).

Over the past decades, there have been a variety of different approaches and methods to characterize and identify different stress granule components and functions (Table 1). A starting point of analysis for any granule-associated client protein is to identify its components with imaging technology. Initially many components of stress granules were identified using specific antibodies via immunofluorescence or immunohistochemistry. These imaging-based experiments detected several different types of stress granule components, including RBPs and translation machinery components. Recent advances in microscopy enable robust live-cell imaging of diffusing granule proteins, providing biophysical data of granule material properties. Fluorescence *in situ* hybridization and the MS2 tagging system can be used for fixed and live tracking of RNAs (Le et al., 2022).

Alternatively, omics-based techniques identify components of the proteome and transcriptome of RNA granules. Previously published work from our lab used ascorbate peroxidase (APEX2) proximity labeling to identify components of SGs in human cells (Markmiller et al., 2018). APEX can also be repurposed to pull down RNAs instead of proteins (Padron et al., 2019). Other techniques such as BioID provide similarly robust datasets of the granule proteome (Youn et al., 2018). We have also pioneered enhanced CLIP technologies, which identify bound RNAs in granules (Van Nostrand et al., 2016; Corley et al., 2020; Blue et al., 2022). Coupling these technologies with different biological conditions can identify stress-responsive changes in granule components, including shifts that are precursors to neurodegeneration (Markmiller et al., 2021).

Biochemical techniques allow the most robust characterization of granules properties and even enable the purification of whole granules from cells. Matheny *et al.* compared two granule isolation methods, and they demonstrated that more SG enrichment comes from differential centrifugation and immunopurification (Matheny et al., 2019). The RNAs from purified granules were similar to the RNAs found in the cellular granules confirmed with FISH. Biophysical techniques such as FRAP and microrheology also identify the material properties of *in vitro* and *in vivo* granules, which may help pinpoint the transition from a normal granule to a disease-associated granule. Overall, the approaches discussed here give a wealth of information for understanding stress granule production and disassembly, function, and regulation, which we anticipate will be important for identifying how and why RNA granules transition into neurodegeneration-linked fibers.

## Conclusion

Sequestering critical RBPs and RNA molecules into granules is an inherently risky maneuver for cells. If a cell does not pause translation, splicing, and other energetically expensive processes, it may not survive the stress event. However, if the stress response persists for too long, the cell may permanently entangle essential machinery for translation, splicing, and other processes into granules. As we discussed above, persistent granules may undergo a phase transition into nondynamic, solid- or gel-like structure, which is a hallmark of neurodegenerative diseases. Therefore, it is necessary to better understand how and why RNA granules mature into nonresolvable aggregates so that we can develop appropriate remedies to counteract deleterious phase transitions.
